# 

**Published:** 1896-06

**Authors:** 


Secretary.

### MISSOURI VALLEY VETERINARY ASSOCIATION.

THE second annual meeting of the Association will be held in Leavenworth, Kansas, Wednesday, June 10, 1896. The meeting will convene at the National Hotel, at 7.30 P.M. Papers will be read by Dr. U. B. McCurdy, Topeka, Kansas, "Tuberculosis;" Dr. N. S. Mayo, Manhattan, Kansas, "Cornstalk Disease;" Dr. R. C. Moore, Kansas City, Mo., "Castration of Ridglings;" Dr. G. C. Pritchard, Topeka, Kansas, "Control of Contagious Diseases in Animals;" Dr. J. O. Young, Concordia, Kansas, "What is Required to Practise Veterinary Science Successfully;" Dr. C. B. McClellan, Lawrence, Kansas (subject not given); Dr. E. H. Biart, Lansing, Kansas, "Preventive Inoculation for Contagious Pleuro-pneumonia."

Election of officers for the ensuing year will also take place, and from present indications we will have a large turnout and a good meeting. Come early and stay late.

S. L. HUNTER, V.S.,
Secretary.

### IMPORTANT MEETINGS IN JUNE.

THE Keystone Veterinary Medical Association of Philadelphia and vicinity will close its most successful series of meetings for years with the gathering on June 9th, at 8 P.M., Broad and Filbert Streets, third floor. A full meeting is earnestly requested. Invitations have been issued to several prominent members of the profession outside of the State. A luncheon will be spread after the close of the regular order of business, which will be shortened on this occasion. This Association announces the adoption of a certificate of membership, and will resume its meetings in September next under the brightest prospects for a number of years. President Hart and Secretary Rhoads are extremely anxious that every member shall be present on June 9th.

THE annual meeting of the Schuylkill Valley Veterinary Medical Association will be held at Hotel Woll, Pottsville, Pa., on Wednesday, June 17th, at 11 A.M. Matters of importance relating to the profession will be discussed. Some interesting papers are promised by Drs. Bieber, "Mange;" Moyer, "Castration;" Faughnan, "Tetanus;" Sallade, "The Social Position of the Veterinarian;" Noack, "Emphysema."

Applications for membership should be addressed to the Secretary.

By order of the President. OTTO NOACK,
Secretary.

### PERSONAL.

Prof. A. Smith, of Toronto, takes an active interest in the Canadian Jockey Clubs, and fills a place on the executive committee for the present year.

Veterinarians Thomas Hodgson and W. W. Stewart filled the professional positions at the recent horse-show in Toronto, Canada.

Dr. J. C. McNeil, of Pittsburg, has been appointed deputy inspector for the Western District of Pennsylvania to represent the State Veterinary Sanitary Board, owing to the prevalence of bovine tuberculosis reported from that district.

Dr. J. S. Butler, of Minneapolis, lectured before the master horseshoers during the month of April.

Dr. D. P. Yonkerman lectured to the master shoers of Kalamazoo, Michigan, during the month of March.

The Pittsburg *Blue-book* contains the name of but one veterinarian.

Dr. A. H. Baker, of Chicago, is on a pleasure-jaunt for a period of two weeks through the sunny South.

The selection of Dr. S. Stewart as dean of the Kansas City Veterinary College, to succeed Dr. Wattles, fully assures the maintenance of the highest purposes of a thorough veterinary education in that school.

Dr. H. Walters, of Wilkesbarre, will conduct the examinations and supervision of the dairies and herds of cattle from which the Hygienic Milk Co., of the above city, will obtain their milk-supply.

Dr. A. B. Campbell has just been appointed milk-inspector for Berlin, Ontario.

Dr. John W. Adams, Professor of Veterinary Surgery and Obstetrics at the Veterinary Department of the University of Pennsylvania, has been appointed consulting veterinarian to the Department of Public Safety in Philadelphia. This is a well-merited appointment, and will reflect the highest credit upon the profession, and give to our city services of a highly valuable character.

Governor Drake, of Iowa, has appointed the following Assistant State Veterinarians in the several counties: S. H. Kingery, Union; R. R. Hammond, Mahaska; J. D. Inger, Bremer; J. H. McLeod, Lloyd; H. A. Fee, Blackhawk; W. H. Austin, Jasper; S. H. Johnson, Carroll; J. W. Scott, Delaware; J. A. Simcoke, J. W. Griffith, Lynn; P. O. Koto, Winnebago; William Benjamin, G. A. Johnson, Woodbury; H. Shipley, O'Brien, E. E. Sayers, Kossuth; and R. G. Rich, Fayette.

Dr. H. D. Hanson, of New York City, has been on a flying trip to St. Louis for rest and recreation.

The Buffalo *Horse World* reports the death of Dr. Hy. Kugler, of Port Chester, N. Y., from glanders contracted while handling an affected horse.

Dr. I. N. Krowl, formerly of Passaic, N. J., is now a resident of Jeffersonville, N. Y.

The Veterinary Department of Cornell University will have a strong faculty to offer to their future students. The New York State Legislature appropriated $25,000 for the first year.

The Ohio State University at Columbus, Ohio, will have in their new agricultural building, among many other facilities, a Pasteurizing room: a room where judging of cattle will be taught.

The many friends of Prof. R. S. Huidekoper will be glad to learn of his continued progress toward restored health and wish for him a prolonged period of rest that will enable him to again resume in the fall the many important positions he has occupied as a teacher the past two years.

Veterinarian G. Howard Davison, of Millbrook, N. Y., is quite an enthusiastic breeder of sheep. He sailed for England in May for the purpose of making some fresh purchases.

